# The effects of case management for breast cancer patients: A protocol for systematic review and meta-analysis

**DOI:** 10.1097/MD.0000000000028960

**Published:** 2022-03-04

**Authors:** Yong Chai, Li Li, Yun-Lian Wu, Tao Wang, Yu-Ming Jia, Xiao-Li Lin, Xi Chen, Hui Zhong, Li-Xia Liu, Lian-De Tao

**Affiliations:** a The Second People's Hospital of Yibin, Yibin, Sichuan, China; b International School of Nursing, Hainan Medical University, Haikou, Hainan, China.

**Keywords:** case management, breast cancer, protocol, systematic review

## Abstract

**Background:**

Female breast cancer is the most common cancer nowadays, and its treatment has a significant impact on patients both physically and psychologically. Many randomized trials have proved that case management (CM) can effectively care for patients. However, there is a lack of systematic scientific evaluation, so this systematic evaluation aims to explore the impact of CM on breast cancer patients.

**Methods:**

PubMed, Embase, Cochrane Library, Scopus, CINAHL were searched. Chinese repositories included China National Knowledge, Infrastructure Database (CNKI), Wan fang Database, China Biology Medicine Database. We will also search unpublished literature at ClinicalTrials.gov. Randomized controlled trials were collected from them. The literature will be screened according to inclusion and exclusion criteria, and 2 researchers will extract the literature independently. The primary outcome indicator for this study will be patient satisfaction. Statistics were performed using RevMan 5.4 software. The quality of each outcome will be evaluated using the Grading of Recommendations Assessment, Development, and Evaluation.

**Results:**

This study will provide the most recent evidence for evaluating the impact of CM on breast cancer patients.

**Conclusion:**

To evaluate the impact of CM on patients with breast cancer.

**Registration number:**

DOI:10.17605/OSF.IO/ZJKHX.

## 1 Introduction

According to statistics, female breast cancer has become the most common cancer in the world, with 2.3 million new cases each year,^[1]^ and the incidence rate of which is showing a younger trend, and the current treatment for breast cancer patients is mainly surgery, supplemented by chemotherapy, radiotherapy, targeted and other treatment modalities,^[2]^ so that patients’ survival time is constantly extended. Breast cancer patients, however, have to endure hematoma, infection, skin flap necrosis, chest wall pain, arm complications, and damage to secondary sexual characteristics caused by surgical treatment in addition to the adverse effects of treatment that other cancer patients will face,^[3–5]^ which creates a general sense of uncertainty in breast cancer patients and causes patients to suffer from anxiety, depression, and other adverse emotions, making them suffer from the double blow of physical and psychological disorders,^[6]^ which has a negative impact on patients’ quality of life and disease regression.

To sum up the need for a new model of care that addresses the individual characteristics and needs of the patient and meets the patient's need for long-term supportive care in the form of rehabilitation guidance, information support, and psychological support has become a new challenge in breast cancer care.

According to the Case Management Society of America, the case management (CM) process is a multidisciplinary and collaborative model that includes assessment, planning, implementation, care coordination, monitoring, and evaluation to improve the quality of care through individualized assessment and rational selection of health care resources to meet the supportive care needs of patients and families.^[7–9]^

CM has now been applied in the disease transition evaluation system for the elderly, which can significantly reduce readmission rates and days in hospital,^[10]^ achieve better results in improving health outcomes in diabetes, chronic obstructive pulmonary disease, and coronary heart disease,^[11]^ and can improve depression levels in cardiac patients to some extent.^[12]^ In breast cancer, studies have shown that patients who participate in CM have lower rates of psychological anxiety and depression and better functional recovery and quality of life of the affected limb after surgery.^[13]^ Some studies have shown that CM can effectively help patients care for their wounds and improve their hospital satisfaction.^[14]^ However, there are limitations in the current CM approaches, which vary in supporting care for breast cancer patients. Some studies have small sample sizes, limited quality, and lack research support from evidence-based medicine, so there is a need to assess the impact of CM on breast cancer patients through systematic evaluation and meta-analysis.

## 2 Objectives

To explore the impact of CM on breast cancer patients and provide evidence-based clinical care support.

## 3 Methods and analysis

### 3.1 Study registration

We are registered at the OSF (https://osf.io/zjkhx). The registration number is DOI: 10.17605/OSF.IO/ZJKHX.

### 3.2 Inclusion criteria

#### 3.2.1 Type of research

(1) All randomized controlled trials (RCTs) which compared CM with other forms of care for breast cancer will be retrieved and recorded, RCTs selected female adults (participants aged >18 years) without regional and language restrictions.
(2) Conference papers, reviews, case reports, animal studies, research protocols, supplementary questions, letters will be excluded.

#### 3.2.2 Type of participants

Female patients with a diagnosis of breast cancer are included. Male patients with breast cancer, patients with metastatic breast cancer, patients with previous mental illness, or impaired consciousness will be excluded.

#### 3.2.3 Type of interventions

The trial group will be patients with breast cancer who have received CM.

#### 3.2.4 Types of comparators

Types of comparators: Patients with breast cancer who received conventional care or other methods of care.

#### 3.2.5 Types of outcome measures

##### 3.2.5.1 Primary outcomes

The primary outcome will be the patient satisfaction.

##### 3.2.5.2 Secondary outcomes

The secondary outcomes will be quality of life, pain, depressive disorder, and anxiety.

### 3.3 Exclusion criteria

Duplicate publications; literature where full text is not available; literature where valid outcomes cannot be extracted; and non-RCT literature.

### 3.4 Study search

The search includes PubMed, Embase, Cochrane Library, Scopus, CINAHL, and Chinese repositories such as China National Knowledge, Infrastructure Database (CNKI), Wan fang Database, China Biology Medicine Database (CBM). We will also search for unpublished literature at ClinicalTrials.gov. The search strategy will be adjusted to the various databases (Table 1).

**Table 1:** The search strategies that will be run in PubMed.

Database	Search strategy
PubMed	#1 “breast Neoplasms”[Mesh]#2 “breast carcinoma∗”[Title/Abstract]#3 “breast tumour∗”[Title/Abstract]#4 “breast cancer”[Title/Abstract]#5 “breast disease”[Title/Abstract]#6 #1 OR #2 OR #3 OR #4 OR #5#7 “Patient Care Management”[Mesh]#8 “Case Management”[Mesh]#9 “Patient Care Planning”[Mesh]#10 “Comprehensive Health Care”[Mesh]#11 “Critical Pathways”[Mesh]#12 ”“Patient Navigation”[Mesh]#13 Disease Management[Title/Abstract]#14 patient navigator[Title/Abstract]#15 “#7 OR #8 OR #9 OR #10 OR #11 OR #12 OR #13 OR #14#16 randomized[Title/Abstract]#17 randomised[Title/Abstract]#18 controlled[Title/Abstract]#19 trial[Title/Abstract]#20 randomized controlled trial[Publication Type]#21 #16 OR #17 AND #18 AND #19 OR #20#22 #6 AND #15 AND #21

### 3.5 Selection of studies

**Figure 1:**
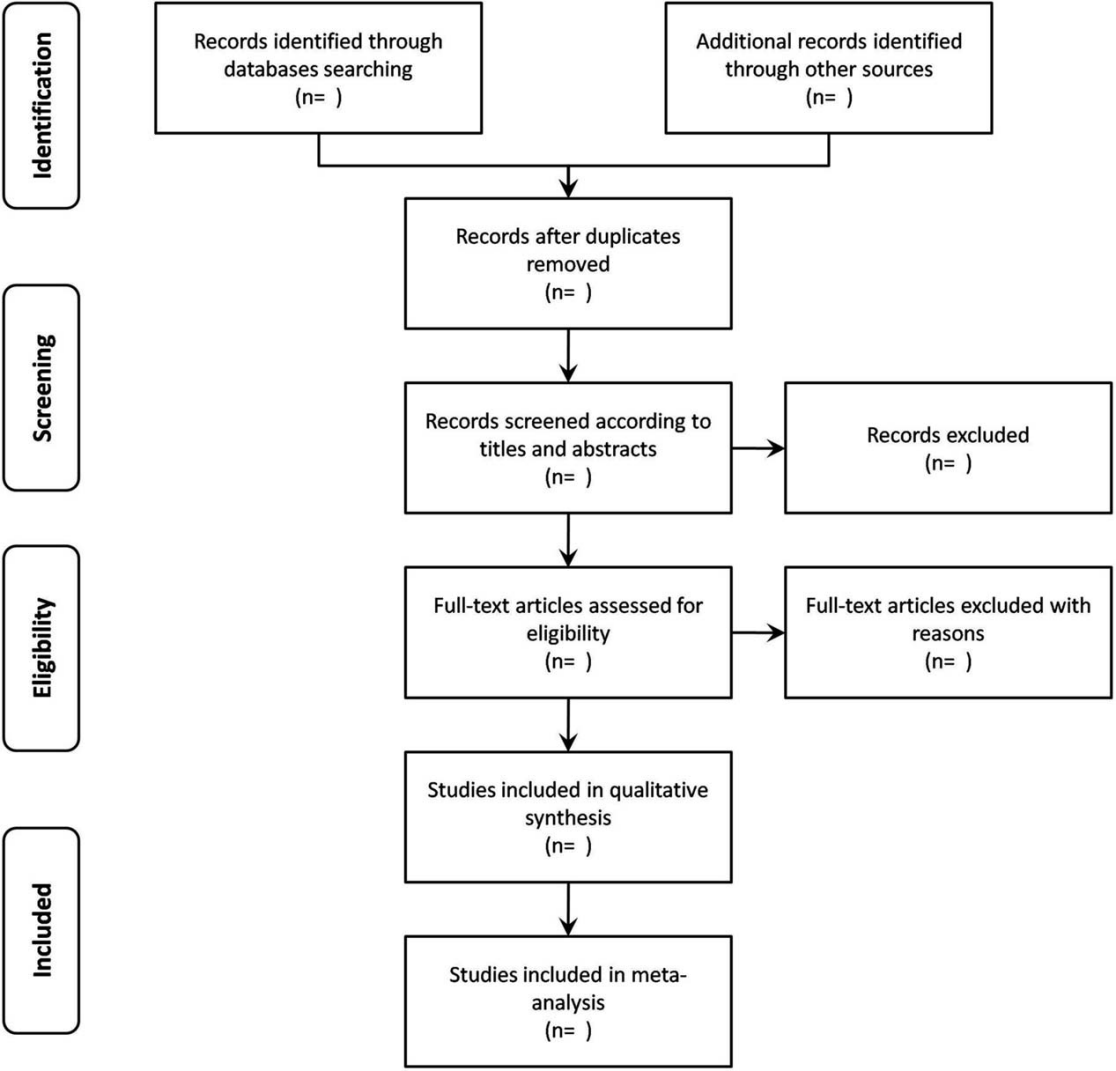
Study selection flowchart.

The literature retrieved from the data will be imported into Endnote software (X9.2, Chandler, AZ). After removing duplicates, 2 researchers (YC and LL) will independently screen the titles and abstracts of the literature based on the inclusion criteria, eliminating those that do not meet the requirements. Then 2 researchers will read the full text of the remaining literature for further screening. The original author and the fourth researcher (XLL) will be contacted for evaluation if necessary. The flow of the study is shown in Figure 1.

### 3.6 Data extraction and management

All data included in the trial were extracted independently by 2 researchers (YC and LL), recorded on a data extraction form and analyzed for: general information (author information, year of publication, country of publication, and funding); trial-type; participant characteristics; interventions; trial outcomes. In a disagreement between 2 researchers, a third researcher (RLW) will arbitrate, and incomplete data will be provided by contacting the original author.

### 3.7 Assessment of the methodological quality

We will use the Cochrane risk assessment tool to assess the quality of the RCT trial literature, which consists of 7 items: random sequence generation, allocation concealment, implementation bias, measurement bias, follow-up bias, reporting bias, and other biases. Each risk bias was judged on the following criteria: low risk of bias, high risk of bias, unclear.

### 3.8 Measures of treatment effect

Data will be analyzed and quantitative data will be synthesized using RevMan V.5.4, London. United Kingdom. Dichotomous variables will be expressed as risk ratios, and continuous variables will be expressed as mean differences or standards mean differences. Evidence-based final effect size estimates and 95% confidence intervals will be given.

### 3.9 Dealing with missing data

In the case of missing data, attempts will be made to contact the authors to obtain them, and if no response is received, these will be clarified and excluded from the literature.

### 3.10 Assessment of heterogeneity

We will use chi-square tests and *I* ^2^ statistics to test for heterogeneity, using *I* ^2^ statistics to determine whether to use a fixed-effects model (*I* ^2^ < 50%) or a random-effects model (*I* ^2^ ≥ 50%), with high heterogeneity being determined when *I* ^2^ > 75% and descriptive analyses will be conducted.

### 3.11 Data synthesis

When the number of included studies exceeds 10, bias will be assessed jointly using funnel plots to observe the symmetry of the funnel plots and to assess the presence of bias.^[15]^

### 3.12 Subgroup analysis

Subgroup analysis will be performed if there is sufficient literature included or a high degree of heterogeneity, depending on the type of patient outcome.

### 3.13 Sensitivity analysis

A sensitivity analysis will be performed using Revman 5.4 software to evaluate the reliability of the meta-analysis. If heterogeneity is high, we will verify the heterogeneity of all included literature 1 by 1, exclude low-quality studies as needed, and then re-run the meta-analysis, comparing the results with the previous meta-analysis. If the results are generally stable, they will be considered reliable.

### 3.14 Grading the quality of evidence

The quality of evidence for all outcomes was graded and recommended using the Grading of Recommendations Assessment, Development, and Evaluation, which classifies the quality of evidence as high, moderate, low, or very low. Evidence from RCT will start at high quality and be selected to be downgraded by 1 or 2 levels depending on risk factors such as the risk of bias, imprecision, inconsistency, indirectness, and publication bias.^[16,17]^ The level of recommendation in Grading of Recommendations Assessment, Development, and Evaluation is divided into strong and weak recommendations based on the level of certainty of the evidence, the desirability of the results, the strength of patient values and willingness, or the cost of resources.^[18]^

### 3.15 Ethics

The study was approved by the hospital ethics committee and did not require ethical approval.

## 4 Discussion

Breast cancer is by far the most common malignancy and the leading cause of death among female oncology patients,^[19]^ and its prevention and treatment is an essential issue in public health. The multiple treatment modalities for breast cancer may lead to patients experiencing pain, infection, and lymphedema, which significantly reduces their quality of life.^[20]^ Numerous care programs have emerged, but few of them, like CM, concentrate on pre-admission assessment, pre-operative guidance, postoperative care, multidisciplinary consultations, psychological support, physical rehabilitation, discharge continuity of care, etc. Although some RCTs have demonstrated that CM can improve the patient experience, there is a lack of systematic scientific evaluation of the impact of CM on breast cancer patients, so this paper aims to provide evidence-based evidence of its effectiveness. This review also has some limitations. however, as differences in the level of CM may lead to heterogeneity, and there may be incomplete results due to the inability to contact the authors.

## Author contributions

**Conceptualization:** Yong Chai, Yun-Lian Wu, Lian-De Tao.

**Data curation:** Yong Chai, Li Li.

**Formal analysis:** Yu-Ming Jia, Xiao-Li Lin.

**Methodology:** Xi Chen, Hui Zhong.

**Software:** Li-Xia Liu.

**Supervision:** Tao Wang.

**Writing – original draft:** Yong Chai, Li Li.

**Writing – review & editing:** Yun-Lian Wu, Lian-De Tao.
